# WGS identifies Lynch syndrome (LS) patients and uncovers a large family with *MSH2*-related LS in Southern Thailand

**DOI:** 10.1371/journal.pone.0348867

**Published:** 2026-05-11

**Authors:** Worrawit Wanitsuwan, Kanet Kanjanapradit, Supakool Jearanai, Yuthasak Suphasynth, Pongsatorn Supaattagorn, Sukanya Vijasika, Surasak Wanram, Chumpol Ngamphiw, Vorthunju Nakhonsri, Sissades Tongsima, Palakorn Satsue, Natnaree Sangkeaw, Sukanya Horpaopan

**Affiliations:** 1 Department of Surgery, Faculty of Medicine, Prince of Songkla University, Songkhla, Thailand; 2 Department of Pathology, Faculty of Medicine, Prince of Songkla University, Songkhla, Thailand; 3 Department of Obstetrics and Gynecology, Faculty of Medicine, Prince of Songkla University, Songkhla, Thailand; 4 Ubon Ratchathani Cancer Hospital, Ubon Ratchathani, Thailand; 5 College of Medicine and Public Health, Ubon Ratchathani University, Ubon Ratchathani, Thailand; 6 Medical Molecular Biotechnology Research Group, National Center for Genetic Engineering and Biotechnology, National Science and Technology Development Agency, Pathum Thani, Thailand; 7 Faculty of Economics, Prince of Songkla University, Songkhla, Thailand; 8 Department of Anatomy, Faculty of Medicine, Chiang Mai University, Chiang Mai, Thailand; Singapore General Hospital, SINGAPORE

## Abstract

This study aimed to investigate Lynch Syndrome (LS)-related germline variants among Southern Thai patients with colorectal cancer (CRC) and other LS-associated cancers who met the revised Bethesda guidelines. A total of 132 probands suspected of LS underwent whole genome sequencing (WGS). Eleven germline variants including pathogenic variants (PVs), likely pathogenic variants (LPVs), and potential variants of uncertain significance (VUSs) in DNA mismatch repair (MMR) genes were identified in twenty-six individuals (20%). Among these, six PVs/LPVs/VUSs were detected in *MLH1*, three in *MSH2*, and two in *PMS2*. Notably, the *MSH2* c.1237C > T PV was detected in ten probands who were determined to share a common ancestry. Subsequent targeted sequencing of 56 relatives revealed fifteen additional carriers, four had already developed CRC or ampullary cancer, while colonoscopy surveillance detected polyps in two others. The benefit-cost ratio (BCR) analysis demonstrated the greater cost-effectiveness of genetic testing compared to endoscopic surveillance to all relatives at risk. Although our cohort is clinically enriched and does not reflect the population prevalence of LS in Southern Thailand, these findings highlight the substantial LS burden within high-risk families and underscore the importance of incorporating genetic screening, counseling, and tailored surveillance strategies into clinical practice.

## Introduction

**Colorectal cancer** (CRC) is one of the leading causes of cancer-related mortality worldwide [1]. In Thailand, a Southeast Asian country, CRC is the only cancer with an increasing incidence in both sexes, ranking as the second most common cancer among females and the third among males, with over 10,000 new cases occurring annually [2,3]. Based on genetics and etiological factors, CRC is typically categorized as sporadic, familial, and hereditary. Sporadic CRC accounts for approximately 80% of cases, with no clear evidence of inheritance. In contrast, around 20% of CRC patients have a positive family history, suggesting a significant genetic predisposition. However, up to 6% exhibit a monogenic inheritance pattern due to highly penetrant germline mutations in cancer driver genes associated with hereditary CRC [4,5].

Lynch syndrome (LS), formerly known as hereditary nonpolyposis colorectal cancer (HNPCC) (MIM#120435), is the most common cancer predisposition syndrome and the most prevalent inherited form of CRC. LS is caused by a heterozygous germline pathogenic variant (PV) in DNA mismatch repair (MMR) genes, including *MLH1*, *MSH2, MSH6,* and *PMS2*, or by deletions in the 5´region of *EPCAM,* leading to epigenetic silencing of *MSH2*. The majority of LS-related PVs (82%) occur in *MLH1* and *MSH2,* the most important predisposing genes for LS, while *MSH6* and *PMS2* account for a smaller proportion (18%) [6].

Although LS predisposes mainly to hereditary CRC and endometrium cancer (EC), it is also associated with a broad extracolonic tumor spectrum, including ovarian, skin, urothelial, small intestine, prostate, brain, and gastric cancers [5,7,8]. The cumulative lifetime risk of CRC and any cancer by age 70 is approximately 34% and 36%, respectively [9].

The hallmarks of LS tumors are the loss of MMR protein(s) expression and microsatellite instability (MSI) resulting from biallelic MMR gene inactivation through a somatic second hit [10]. These parameters are routinely employed for tumor screening in suspected LS cases, and in several Western countries, are recommended as part of standard diagnostics for all CRC and EC patients [11]. Clinical suspicion of LS warrants genetic testing based on the revised Bethesda guidelines (RBG) [12] and/or Amsterdam Criteria II (ACII) [13]. Identifying individuals with LS enables risk stratification, early cancer detection, and the implementation of targeted surveillance strategies, ultimately reducing CRC-related morbidity and mortality [14].

In the Western population, LS is the most common hereditary (monogenic) CRC syndrome, accounting for 2–3% of all CRC [15–17]. The prevalence of LS-related germline PVs/LPVs in that population ranges from 1:300–1:500 [18–20]. However, in Thailand, there is no standardized procedure for hereditary cancer screening, and as most studies do not specifically focus on hereditary cancers [21], the prevalence of LS remains unknown. Without systematic screening or genetic testing programs, many cases may go undiagnosed, leading to an underestimation of true LS prevalence although it is presumed to be similar to other populations with comparable genetic backgrounds.

The genomic medicine era has continuously grown worldwide, and in Thailand, the National Genomics Thailand Project began in 2020. Thus, to manage patients and their families effectively, comprehensive preparation across all aspects of genomic and precision medicine such as infrastructure, workforce training, ethical frameworks, etc., is essential [22,23].

The primary objective of this study was to identify germline PVs/LPVs in patients suspected of LS enrolled in the National Genomics Thailand Project. Interestingly, we identified that ten out of 132 patients carried the same *MSH2* variant, and we verified that they were unexpectedly related by ancestry. Subsequently, we successfully conducted genetic counseling and testing on family members, identifying fifteen out of 56 relatives who carried the same *MSH2* variant. This finding underscores the critical role of genetic counseling and germline PVs/LPVs screening in the early identification of at-risk individuals. These strategies are essential for personalized cancer prevention, guiding resource allocation, and shaping national health policies to mitigate the socioeconomic impact of hereditary CRC.

## Materials and methods

### Patients and samples

This study was conducted in accordance with the Declaration of Helsinki and was approved by the Human Research Ethics Committee (HREC), Faculty of Medicine, of the Prince of Songkhla University (PSU), Thailand (REC.64-223-10-1; approval date: January 5, 2022) and the ethical committee of Ubon Ratchathani Cancer Hospital (URCH), Thailand (EC 013/2021; approval date: October 20, 2021). Participant recruitment took place between January 29, 2022, and December 29, 2023. Due to the necessity of accessing clinical data, family history, and histopathological information, the authors WW, NS, and PS could access information that could identify individual participants during and after the data collection process.

All patients and recruited family members were informed of their inclusion in the registries, and written informed consent was obtained during genetic counseling sessions. The individual in this manuscript has given written informed consent to publish these case details. For participants aged 18 years and above, written informed consent was obtained directly. For participants under 18 years of age, written informed consent was obtained from their parents or legal guardians, and written assent was obtained from the participants themselves. All minors were accompanied by their parents or guardians during the counseling sessions, and an information letter explaining the study’s purpose was provided. There were no deviations from the approved study protocol

Initially, a total of 132 patients with familial CRC and/or EC and with/without other cancer-related LS diagnosed and treated at the Songklanarind Hospital or Ubon Ratchathani Cancer Hospital between October 2001 and December 2023 were recruited for the study. All of them met at least one criteria of the RBG which were younger than 50 years when the cancer was detected, and/or had synchronous/metachronous CRC or other LS-associated tumors at any age, and/or having first- or second-degree relatives with CRC or LS-associated tumors diagnosed at an early age or in at least two family members [12]. Their histopathological demographics such as age at diagnosis, tumor site, staging, histologic grade, type, and metastasis, were collected from the Hospital Information System (HIS).

### DNA extraction from blood and QC

Genomic DNA was isolated from peripheral blood lymphocytes using the GeneJET Genomic DNA Purification Kit (Thermo Scientific, Lithuania) following the manufacturer’s instructions. The extracted DNA was qualified and quantified using a Nanodrop One fluorescence spectrophotometer (Thermo Scientific, USA) and stored at 4°C until used.

### DNA extraction from FFPE tissue

Tumor DNA was extracted from formalin-fixed paraffin-embedded (FFPE) tumor QIAamp Tissue Kit or the Reliaprep FFPE gDNA Miniprep System (Promega) according to the manufacturer’s recommendations. DNA concentration was measured by a Nanodrop One fluorescence spectrophotometer (Thermo Scientific, USA), and samples were stored at 4°C.

### Immunohistochemical staining of MLH1, PMS2, MSH2, and MSH6 proteins

Immunohistochemical (IHC) staining of DNA mismatch repair (MMR) proteins including MLH1, PMS2, MSH2 and MSH6, was performed using mouse monoclonal MLH1, MSH2, MSH6 and PMS2 antibodies (Abcam, USA). Expression of MMR proteins in the nucleus of tumor cells was defined as positive staining of these proteins. Normal expression of protein was defined as the presence of nuclear staining in colon cancer cells. Loss of staining in carcinoma with concurrent positive staining in nuclei of normal colon epithelial cells indicated absent expression of protein.

### Microsatellite analysis

Analysis for microsatellite instability (MSI) was performed with the automated Idylla™ MSI test prototype cartridges using seven microsatellite markers (ACVR2A, BTBD7, DIDO1, MRE11, RYR3, SEC31A, and SULF2). Following the manufacturer’s protocol, sections of 10-μm thickness were obtained from formalin-fixed paraffin-embedded (FFPE) tissue samples, covering an area of 100–200 mm². Each section contained at least 20% tumor cells. The prepared tissue sections were then placed into a sample cartridge, which was subsequently inserted into the instrument as per the previously established method [24]. The Idylla™ Console software analyzed results and generated a final report of microsatellite status as microsatellite stable (MSS) or microsatellite instable (MSI).

### Whole genome sequencing and bioinformatic pipelines

Genomic DNA extracted from peripheral blood lymphocytes of 132 suspected LS patients underwent whole genome sequencing (WGS) on the DNBSEQ-T7 platform (MGI Tech Co.). The MGI MGIEasy FS PCR-Free DNA Library Prep Set was used to prepare DNA libraries. Reads (>100 bp) with Q20 > 90% and Q30 > 80% were aligned to GRCh38 with decoy genomes using the Burrows-Wheeler Aligner (BWA) [25]. Quality control with SAMtools and GATK CollectWGSMetrics required mapping rate ≥ 90%, unique mapping ≥ 85%, mean depth > 30x, > 90% of bases at ≥ 20x, and > 95% of bases at ≥ 10x. Across LS–associated genes; *EPCAM* (NM_002354.3), *MSH2* (NM_000251.3), *MSH6* (NM_000179.3), *MLH1* (NM_000249.4), and *PMS2* (NM_000535.7), the mean depth was 38x with 86–96% of target regions covered at ≥20x, supporting high-confidence variant calls (S1 Table). Short variants (SNPs and INDELs) were called following GATK4.0 best practices [26].

Duplicates were marked with Picard tools, followed by base quality score recalibration (BQSR) and local realignment around indels. Variants were called using GATK HaplotypeCaller, and filtered using VQSR/VariantFiltration. High-quality variants were annotated with Ensembl Variant Effect Predictor (VEP) build 113 [27] using dbNSFP 4.9a [28] and gnomAD v 4.1 [29]. TAPES [30], was used to classify variants as pathogenic (P), likely pathogenic (LP), variant of uncertain significance (VUS), likely benign (LB), or benign (B) according to the 2015 ACMG/AMP guidelines [31]. Sample-level annotated variants are available through the Thailand Variant Annotation and Prioritization Platform (v@pp) (https://vapp.genomicsthailand.com/), which provides direct link-outs to curated knowledge bases including GeneCards, OMIM, GeneReviews, gnomAD, and VarSome.

Structural variants (SVs) were called from BAM files using Manta [32] and annotated with AnnotSV [33]. We focused on *EPCAM, MSH2*, *MSH6*, *MLH1*, and *PMS2,* and classified variants by pathogenicity (P > LP > VUS), according to the 2015 ACMG/AMP guidelines. All variants were cross-referenced with InSiGHT, ClinVar, and gnomAD databases.

For promising VUSs, pathogenicity was assessed using in-silico tools, including PolyPhen-2, Mutation Taster, SpliceAI, and Priors. We further applied the preprinted ClinGen InSiGHT Hereditary Colorectal Cancer/Polyposis Variant Curation Expert Panel (VCEP) specifications for MMR genes v 1.0.0 to refine variant classification where possible.

### *MSH2* c.1237C > T genotyping study

The genomic DNA of family members was extracted from peripheral blood leukocytes using the GeneJET Genomic DNA Purification kit (Thermo Scientific, Lithuania) according to the manufacturer’s instructions. DNA samples were stored at −20°C until used. Primers were designed with Primer3web version 4.1.0 (https://primer3.ut.ee/); the sequences of forward and reverse primers were 5′- AAGATGCAGAATTGAGGCAGA-3′ and 5′- GGACAGCACATTGCCAAGTA-3′, respectively. The expected PCR product size was 242 bp. PCR was performed following standard protocol. Briefly, the PCR mixtures contained 10 μl of 2X PCR SuperMix (Bio-Helix Co. Ltd.), 1 μl of each primer at 10 μM, 8 μl of distilled water, and 1 μl of the DNA template, a total volume of 20 μl for each reaction. The reactions were conducted in the C1000 Thermal Cycler (BioRad Laboratories, USA) under the following conditions as initial denature of 95^o^C for five minutes, followed by five cycles of denaturation at 95^o^C for 30 sec, annealing at 60^o^C for 30 sec, and extension at 72^o^C for one minute, and then a final extension at 72^o^C for five minutes. After amplification, 2 μl of each PCR product was visualized by gel electrophoresis in a 1.5% agarose gel mixed with RedSafe™ Nucleic Acid Staining Solution (iNtRON Biotechnology). PCR products were purified using NucleoSpin Gel and PCR Clean-up Kit (Macherey-Nagel GmbH and Co. KG, Düren, Germany). The purified PCR products were bidirectionally sequenced using the Sanger method at Macrogen Co. Ltd. (Seoul, Korea).

### *BRAF* V600E and *KRAS* G12D analyses

To examine the *BRAF* V600E and *KRAS* G12D variants in tumor DNAs, we performed PCR and direct sequencing following standard protocol as described above. The 228 bp fragment of *BRAF* encompassing exon 15 and/or 225 bp fragment of *KRAS* encompassing exon 1 were amplified using 2X PCR SuperMix (Bio-Helix Co., Ltd.) and primers *BRAF* forward 5’-TGCTTGCTCTGATAGGAAAATG-3’ and reverse 5’-AGCATCTCAGGGCCAAAAAT-3’, and/or primers *KRAS* forward 5’-TTAACCTTATGTGTGACATGT TCTAA-3’ and reverse 5’-AGAATGGTCCTGCACCAGTAA-3’. PCR product was purified using Purelink™ Genomic DNA Mini kit (Invitrogen) and then submitted to Macrogen’s sequencing service (Macrogen Co. Ltd.) for the sequencing.

### Screening of family members of probands with *MSH2* PV and segregation analysis

The identity-by-descent (IBD) was performed by using KING software [34] to identify the relatedness among these patients. Genetic counselling was offered to family members of the probands who carried an MMR PV/LPV. Genetic testing was carried out in high-risk relatives with/without previous cancer diagnosis if they consented. Particularly, the family members of probands with *MSH2* c.1237C > T, we contacted them by telephone and invited them to the hospital for interviewing in order to obtain their family histories. Their clinical data was retrieved from the Hospital Information System (HIS). A detailed family history and pedigree chart were created after interviewing to see whether the segregation of the PV corresponds with the LS-associated cancers.

### Penetrance rate and cumulative risk calculations

Given the small number of cohort, a crude penetrance was calculated as a simple proportion of carriers who had any LS-associated cancer divided by the total number of carriers. To obtain more refined estimates, cumulative risk was calculated using AI-assisted computation (ChatGPT, OpenAI) in conjunction with actuarial life-table analysis with 10-year age intervals, following previously established approaches for hereditary cancer risk estimation [35–37]. Sex-stratified life tables were subsequently constructed by generating separate age-specific risk sets for males and females, with parallel calculation of interval-specific probabilities, survival functions, cumulative risks [35–37].

### Cost–benefit analysis

Cost–benefit analysis is an approach used to evaluate whether a project, policy, or investment is worthwhile. It compares all expected costs (monetary and non-monetary) against all expected benefits, usually over time, to determine if the benefits outweigh the costs. Cost–Benefit Analysis begins by clearly defining the project or decision and setting its scope and objectives. Next, all potential costs and benefits are identified, including direct, indirect, tangible, and intangible factors. These are then quantified in monetary terms, often using financial proxies for non-market values. Since money has different values over time, future costs and benefits are discounted to present values using a discount rate. To evaluate economic viability, decision criteria like the Benefit–Cost Ratio (BCR) are computed. Finally, conclusions are drawn, and recommendations are made based on whether the benefits outweigh the costs. Key indicators used to consider whether the benefits outweigh the costs are as follows:

Benefit–Cost Ratio (BCR): Ratio of present value of benefits to present value of costs.


$$BCR=\frac{\sum \frac{Benefits_{t}}{(\text{1}+r{)}^{t}}}{\sum \frac{Costs_{t}}{(\text{1}+r{)}^{t}}}$$

Decision rule: If BCR > 1, project is economically justified, whereas BCR < 1, costs outweigh benefits.

## Results

### Clinicopathological data of 132 suspected Lynch syndrome patients

A total of 132 suspected Lynch syndrome (LS) patients (56 male and 76 female) who were diagnosed with colorectal cancer (CRC) and/or endometrial cancer (EC) and with other cancer-related LS at Prince of Songkhla University and Ubon Ratchathani Cancer Hospital between 2001 and 2023 were recruited in the study. The age of onset was 16–93, with the average age at 52. All patients met the RBG [12] but only 59 patients (45%) fulfilled the ACII [13]. While the majority of patients (92%) developed CRC with/without extracolonic cancer (average age of 52), the minority of them (8%) developed EC with/without OC (average age of 50). Sixty-one patients (46%) were younger than 50 years, thirty-one patients (24%) were 50–59 years, twenty-nine patients (22%) were diagnosed at 60–69 years, and eleven patients (8%) were older than 70 years at the age of diagnosis (Table 1).

**Table 1:** Clinicopathological features of 132 suspected LS patients.

Patients’ characteristics (total 132)	Number of patients/tumors (%)
**Sex**	
Male	56 (42.4%)
Female	76 (57.6%)
**Age**	
< 50 yr	61 (46.2%)
50–59 yr	31 (23.5%)
60–69 yr	29 (22%)
≥ 70 yr	11 (8.3%)
[min-max, mean ± SD (yr)]	[16-93, 52.1 ± 13.5]
**Primary cancer**	
CRC	110 (83.3%)
CRC and other cancers (prostate, duodenum, cervix, breast, ampulla of Vater, pancreas, soft tissue, EC, OC)	12 (9.1%)
EC	10 (7.6%)
**Tumor location of CRC** (122 patients)	
Right-sided	16 (13.1%)
Left-sided	89 (73%)
N/A	17 (13.9%)
**Histopathological type of CRC** (122 patients)	
Adenocarcinoma	108 (88.5%)
Hyperplastic polyps	4 (3.3%)
N/A	10 (8.2%)
[min-max, mean ± SD (yr)]	[16-93, 52.3 ± 13.9]
**Histopathology report of EC** (13 patients)	
Adenocarcinoma	9 (69%)
Carcinoma	4 (31%)
[min-max, mean ± SD (yr)]	[35-67, 49.8 ± 9.4]
**Microsatellite status**	
MSI	18 (13.6%)
MSS	43 (33%)
N/A	71 (53.4%)
**Loss of MMR protein expression**	
MLH1/PMS2/MSH2/MSH6: -/-/ + /+	10 (7.6%)
MLH1/PMS2/MSH2/MSH6: + / + /-/-	6 (4.5%)
MLH1/PMS2/MSH2/MSH6: + / + / + /+	10 (7.6%)
N/A	106 (80.3%)

-, loss of expression; + normal expression; CRC, colorectal cancer; diff, differentiated; EC endometrial cancer; MMR, mismatch repair; MSI, microsatellite instable; MSS, microsatellite stable; N/A, not available; OC, ovarian cancer; SD, standard deviation; yr, year.

### Analysis of microsatellite status and immunohistochemical staining of DNA MMR proteins

The microsatellite instability (MSI) test was performed in 61 of 132 suspected LS patients. Eighteen of them exhibited MSI-High (MSI-H), while 43 were MSS. Of the 18 MSI-H cases, eight showed loss of MLH1 and PMS2 expression, and five showed loss of MSH2 and MSH6 expression. For the remaining five MSI-H patients, the MMR IHC staining result was unavailable. Of the 43 MSS cases, IHC staining of MMR proteins was performed in eight cases. All of them showed normal expression of all four MMR proteins, consistent with their MSS status (Table 2).

**Table 2:** Number of patients with microsatellite status and expression analysis of MMR proteins.

Microsatellite status	MLH1 & PMS2 loss	MSH2 & MSH6 loss	All intact	Unknown	Total
**MSI-High**	8	5	0	5	18
**MSS**	0	0	8	35	43
**Unknown**	2	1	2	66	71
**Total**	10	6	10	106	132

MSI, microsatellite instable; MSS, microsatellite stable.

Of the 71 cases who did not undergo MSI testing, IHC staining was done in five cases. Two showed loss of MLH1 and PMS2 expression, one showed loss of MSH2 and MSH6 expression, and one exhibited normal expression of all four MMR proteins.

### Identification of germline pathogenic and likely pathogenic variants in DNA MMR genes in 26 of 132 suspected LS patients

Analysis of *MLH1, MSH2, MSH6, PMS2*, and *EPCAM* identified 8 PVs/LPVs in 23 suspected LS patients; five variants in *MLH1* (11 patients), two in *MSH2* (11 patients), and one in *PMS2* (1 patient). In addition, three promising VUSs were detected in MMR genes (Table 3). In total, 26 patients carried a PV/LPV/promising VUS. Among them, twelve with *MLH1* variants were diagnosed between 23–65 years (mean ± SD: 49.0 ± 12.5), twelve with *MSH2* variants between 23–71 years (mean ± SD: 44.5 ± 12.2), and two patients with *PMS2* variants at 47 and 65 years (mean 56). No PV/LPV/promising VUS was detected in *MSH6* or EPCAM.

**Table 3:** Clinicopathological characteristics of 26 suspected LS patients carrying PVs/LPVs/promising VUSs in DNA MMR genes.

Variant	No. of patient (%)	Patient ID	Sex	Age at Dx (year)	Dx	Localization	Tumor grade/ Stage	Pathology report	Microsatellite status	IHC MLH1/PMS2/ MSH2/MSH6	BRAF V600E/ KRAS G12D
***MLH1* (NM_000249.4)**											
c.1546del, p.Gln516ArgfsTer19	1 (0.7%)	GN024	M	52	CRC	Ascending colon	T2N0M0	Adenocarcinoma	N/A	N/A	wt/wt
c.1673_1676dup, p.Phe560ThrfsTer9	3 (2.2%)	GN020	F	65	CRC	N/A	N/A	Adenocarcinoma invades pericolic fat	N/A	N/A	N/A
		GN025	M	39	CRC	N/A	pT4bN2M0/ IIB	Adenocarcinoma, well diff	MSI-H	-/-/ + /+	wt/wt
		GN092	F	57	CRC	N/A	T2N1M0	Adenocarcinoma	N/A	N/A	N/A
c.350C > T, p.Thr117Met	1 (0.7%)	GN046	F	59, 71	CRC	Ascending colon, descending colon	T2N0M1	Adenocarcinoma	MSI-H	N/A	wt/wt
c.381-1G > C, splice acceptor	2 (1.4%)	GN082	F	55	EC	Corpus uterus	IB G2	Adenocarcinoma	MSI-H	-/-/ + /+	N/A
		GN098	M	48	CRC	Descending colon	T3N0M0/ II	Adenocarcinoma	MSI-H	N/A	wt/wt
c.884 + 3A > G, splice donor	4 (2.9%)	GN026	M	23	CRC	Descending colon	-/ III	Adenocarcinoma, well diff	MSI-H	-/-/ + /+	wt/wt
		GN050	F	46, 50	CRC, OC	Caecum	-/ II	CRC: Carcinoma	MSI-H	-/-/ + /+	wt/wt
		GN062	F	42, 44	CRC, EC	N/A	-/ IIIA	CRC: Adenocarcinoma; EC: Endometroid adenocarcinoma grade 3	N/A	-/-/ + /+	N/A
		GN184	M	37	CRC	N/A	T4N2M0/ III	Mucinous adenocarcinoma with signet ring features	MSI-H	-/-/ + /+	wt/wt
c.1883T > C*, p.Leu628Ser	1 (0.7%)	GN065*	M	65, 68	Duodenal Ca, CRC	N/A	pT3N0M0/ IIIB	CRC: Adenocarcinoma, well diff	N/A	-/-/ + /+	wt/wt
***PMS2* (NM_000535.7)**											
c.1738A > T, p.Lys580Ter	1 (0.7%)	GN236	M	47	CRC	Sigmoid colon	N/A	Adenocarcinoma, well diff	N/A	N/A	N/A
c.4dup*, p.Glu2GlyfsTer4	1 (0.7%)	GN053*	M	65, 70	CRC, prostate Ca	Rectum	T3N0M0/ I (Dworak system)	Adenocarcinoma both	N/A	N/A	N/A
***MSH2* (NM_000251.3)**											
c.923_937del*, p.Arg308_Phe313delinsIle	1 (0.7%)	GN048*	F	41, 47	EC, CRC	Ascending colon	T3N0M0/ II	CRC: Adenocarcinoma; EC: carcinoma grade 2	MSI-H	+/ + /-/-	N/A
c.1405del, p.Val470Ter	1 (0.7%)	GN141	F	35	CRC	N/A	T3N0M0	Adenocarcinoma, well diff	MSI-H	+/ + /-/-	wt/wt
c.1237C > T, p.Gln413Ter	10 (7.2%)	GN007	F	47	Polyps, EC	Corpus uterus	IB G2	Endometrioid adenocarcinoma	MSI-H	N/A	N/A
		GN012	F	47	EC	Corpus uterus	IB G2	Endometrioid adenocarcinoma	MSI-H	+/ + /-/-	wt/wt
		GN013	M	30	CRC	Ascending colon	T3N0M0/ II	Adenocarcinoma, well diff	N/A	N/A	N/A
		GN027	M	23	CRC	Rectum	T3N0M0/ I (Dworak system)	Adenocarcinoma, poorly diff	MSI-H	+/ + /-/-	wt/wt
		GN064	F	35	EC	Corpus uterus	IB G2	Endometrioid adenocarcinoma	MSI-H	+/ + / + /+	wt/wt
		GN073	M	47	CRC	Transverse colon	T1N0M0/ I	Adenocarcinoma, well diff	N/A	N/A	N/A
		GN085	F	58	CRC	Ascending colon	T3N1M0/ III	Adenocarcinoma, well diff.	N/A	N/A	N/A
		GN097	F	45	EC	Corpus uterus	IB G2	Endometrioid adenocarcinoma	N/A	N/A	N/A
		GN197	M	46	CRC	Descending colon	T4N0M0/ II	Mucinous adenocarcinoma, well diff.	N/A	+/ + /-/-	N/A
		GN360	M	70	CRC	Ascending colon	T4N0M0/ II	Mucinous adenocarcinoma, moderately diff.	N/A	+/ + /-/-	N/A

*, promising variant of uncertain significance (VUS); + , normal expression; – loss of expression; Ca, cancer; CRC, colorectal cancer; diff, differentiate; Dx, diagnosis; EC, endometrial cancer; F, female; IHC, immunohistochemical; M, male; MMR, mismatch repair; MSI-H, microsatellite instability-high; N/A, not available; OC, ovarian cancer; wt, wild type.

Of the three promising VUSs, *MLH1* c.1883T > C; p.Leu628Ser occurred in a patient (GN065) who developed duodenal cancer and CRC at the ages of 65 and 68 respectively. Pathological study showed loss of *MLH1* & *PMS2* expression and *BRAF* V600E negativity. Referring to his family history, his three brothers developed CRC at the ages of 30, 40, and 50. All of them are deceased. In addition, a brother of his mother had also developed CRC (unknown age) and was deceased. This VUS is absent from gnomAD. It is reported once on InSiGHT as it was found in one LS patient who had a positive family history and twice on ClinVar; one was found in an LS patient and the other was reported with an unknown phenotype. Since segregation of the variant with the disease could not be verified, the current evidence does not meet the criteria to classify it as PV/LPV although the pathological findings and family history support a likely association.

*MSH2* c.923_937del; p.Arg308_Phe313delinsIle, detected in a female patient (GN048) with early-onset CRC and EC at 41 and 47 years with MSI-H and loss of MSH2 and MSH6 expression, was absent from InSiGHT, ClinVar, and gnomAD and classified as VUS under the ClinGen InSiGHT Hereditary Colorectal Cancer/Polyposis VCEP MMR specifications because in-frame length change alone is not considered sufficient for LPV classification without functional or segregation data.

*PMS2* c.4dup; p.Glu2GlyfsTer4, a frameshift variant was found in a male patient (GN053) who developed rectal cancer and prostate cancer at the ages of 65 and 70, respectively. This germline variant is absent from gnomAD and ClinVar, but reported once on the InSiGHT database, found in a breast cancer patient [38]. The Franklin, an online variants classification tool (https://franklin.genoox.com/clinical-db/home), based on the 2015 ACMG/AMP guidelines [31], classified this variant as a LPV (PVS1, PM2). Consistent with the Franklin classification, we classified this variant as a PV based on the following criteria: 1) it is a frameshift, protein length changing variant (PM4); 2) loss of function is a well-established disease mechanism for this gene (PVS1); and 3) it is absent from gnomAD, the population database (PM2).

Among *MLH1* PV/LPV carriers, most developed CRC (92%) with one extracolonic tumor (8%). In contrast, among *MSH2* PV/LPV carriers, both CRC and extracolonic cancers occurred with comparable frequency (Table 3).

Structural variants (SVs) were detected with Manta and annotated using AnnotSV (S2 Table). A 79-bp deletion in *MSH2* was observed in 111 patients (47 heterozygous, 64 homozygous), and a separate 80-bp deletion in *MSH2* was found in one patient. In *MLH1*, a 56-bp deletion was identified in two patients. All deletions were intronic and classified as VUS.

### Family histories and pedigree analysis of patients with *MSH2* c.1237C > T, p. Gln413Ter reveal one big family tree in Phatthalung province

**Fig 1:**
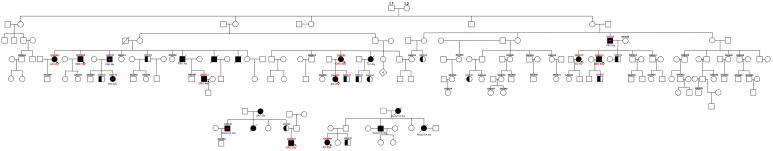
Pedigree of the nine probands with *MSH2* c.1237C > T, p.Gln413Ter; seven probands (GN007, GN012, GN013, GN073, GN085, GN097, and GN360) are from the same family in Phatthalung Province, and two probands (GN027 and GN064) are in some way relatives. The pedigree of the proband GN197 is not shown.

In our study, *MSH2* c.1237C > T PV emerged as the most frequent finding. The IBD analysis demonstrated genetic relatedness among seven patients carrying this variant. Specifically, GN085 and GN007 were identified as a parent-offspring pair, GN012 and GN013 as full siblings, and GN097 and GN360 as full siblings as well. GN073 was found to be a second-degree relative of GN097 and GN360. (S3 Table). We contacted the probands and/or their family members. Based on the genetic counselling, we identified that the seven probands are originally from Phatthalung province and they share the ancestors (I.1 and I.2) (Fig 1). Patient GN027 was found to have the same last name as individual I.2. His maternal grandfather was born in Phatthalung, and he vaguely recalled being a cousin of the extended family, although he could not confirm whether the exact relationship is from the paternal or maternal side. Similarly, patient GN064 reported that her ancestors originally resided in Phatthalung but migrated to Songkhla during her great-grandparents’ or great-great-grandparents’ generation. In contrast, patient GN197 had no identifiable connection to this extended family. None of his family members has developed cancer. Additionally, their ancestors did not reside in Phatthalung but originated from Songkhla.

Patient IDs written in red with the arrow represent the probands; Patient IDs written in black represent the family members who were tested but are not carriers; Patient IDs written in purple represent the family members who were tested and carry the *MSH2* PV.

### Histopathological studies of 10 suspected LS patients with *MSH2* c.1237C > T PV

Of the ten patients, six (4 male, 2 female) developed CRC at the ages of 23, 30, 46, 47, 58, and 70, and four female patients developed EC at the ages of 35, 45, 47, and 47 (mean age ± SD: 44.8 ± 13.4). Four patients had MSI-H, whereas the microsatellite status was unknown to the other six patients (Table 3).

### Pathogenicity classification of *MSH2* c.1237C > T, p.Gln413Ter

The *MSH2* c.1237C > T, p.Gln413Ter has already been reported as PV three times (1 LS, 2 unknown) on the InSiGHT database and reported six times (5 PVs, 1 LPV) on ClinVar (https://www.ncbi.nlm.nih.gov/clinvar/ variation/218030/). Three of the five PVs were identified in LS while the other three reports did not provide the condition of the patients. The *MSH2* c.1237C > T, p.Gln413Ter is not reported on gnomAD.

### Germline variant screening for *MSH2* c.1237C > T in family members

After interviewing and genetic counseling, we were able to collect venous blood from 56 family members for an analysis of the germline *MSH2* c.1237C > T variant (Fig 1). By sequencing, we can identify *MSH2* c.1237C > T in 15 members (27%) (5 females, 10 males). The ages of carriers were between 13 and 84 years (mean ± SD: 44 ± 14.6). A review of the medical history revealed that one male (GN109) developed ampullary cancer at the age of 73, while two members; one female (GN415) and one male (GN498) developed CRC at the age of 33 and 52 years, respectively. During a surveillance program, we detected an early-stage CRC in a 55-year-old male, and we found polyps in two members (ages 34 and 58 years). The other nine members; 5 males (GN014, GN478, GN504, GN530, GN637), and 4 females (GN028, GN029, GN037, GN505) with the age of 34–65 years (mean ± SD: 47 ± 12.5) were still healthy. Nonetheless, the 13-year-old boy (GN637) has not undergone colonoscopy.

Referring to patient GN027, who vaguely recalled being a cousin of the main family, the *MSH2* sequencing showed that he inherits *MSH2* c.1237C > T from his carrier mother GN028 (49 years old). The PV is likely from his maternal grandmother who developed CRC at the age of 59. She also passed this PV to his son GN498 who developed rectal cancer at the age of 52.

### Penetrance rate analysis and cumulative risk assessment

Among 25 *MSH2* carriers, 10 probands and 15 family members, 14 had been diagnosed with either colorectal cancer (CRC), endometrial cancer (EC), or Ampulla of Vater cancer. While two of them have had polyps, the other nine members were still healthy at the time of surveillance. Therefore, the penetrance rate was simply calculated to be 56%.

Cumulative risk was assessed by stratifying individuals into age groups and calculating the progressive probability of developing CRC or EC over time. The results show that the cumulative risk of CRC or EC increases steadily with age, reflecting the age-related nature of cancer incidence. The cumulative risk analysis revealed a progressive increase in the probability of developing CRC or EC with age, reaching 30% by age 20–29, 63% by age 40–49, and 100% by age 60–69. This suggests that nearly all individuals who developed CRC or EC in the dataset were diagnosed by their late 60s. Additionally, the analysis showed that females had a higher cumulative risk than males. The cumulative risk in females reached 100% by age 50–59 years while males reached 100% cumulative risk at 60–69 years, indicating a later onset compared to females.

### Economic analysis

Following standard clinical guidelines, ten probands and their 56 family members would require annual endoscopic surveillance. Based on the Familial Adenomatous Polyposis (FAP) model (34 endoscopies for seven family members), we estimated an average of five endoscopies per person by age 25 [39]. Assuming each undergoes five endoscopies at 2,300 THB per session, the total endoscopic surveillance costs 759,000 THB (approximately 23,000 USD). The total surveillance expenditure would be substantial. Genetic testing costs include 490,000 THB (49,000 THB/proband) for ten probands and 11,200 THB (200 THB/person) for targeted mutation testing across 56 family members, resulting in a total cost of 501,200 THB. The analysis demonstrates that genetic testing achieves a 34% cost reduction (257,800 THB) compared to endoscopy-based surveillance. Furthermore, genetic testing generated indirect benefits through early detection and prevention. Avoided late-stage CRC treatment costs included early polyp removal (two cases), early-stage CRC treatment (one case) and late-stage CRC treatment (two cases). The polyp removal cost varies depending on the polyp size and treatment technique. The average cost of polyp removal is 20,000 THB. Early-stage treatment costs approximately 400,000 THB/person. Late-stage CRC treatment costs an average of 500,000 THB/person (minimum 1,000,000 THB for two cases, including surgery, chemotherapy, and hospitalization) [40]. Combined, these indirect benefits total 1,440,000 THB (43,600 USD).

It is unnecessary to discount all benefits and costs at the present value because they are assumed to occur in the same period. Therefore, the BCR equals 3.39, calculated by dividing the sum of direct (257,800 THB) and indirect benefits (1,440,000 THB) by genetic testing costs (501,200 THB). This ratio indicates that each 1 THB invested in genetic testing generates 3.39 THB in economic returns, thus demonstrating high cost-effectiveness. Additional indirect benefits that have not been monetized or incorporated into the BCR calculation, such as recovery from CRC, improving the quality of life of patients and their families.

## Discussion

Germline pathogenic variants account for up to 5% of inherited colorectal cancers, of which up to 3% cause Lynch syndrome (LS). This study primarily aimed to identify pathogenic variants in suspected lynch syndrome patients to be able to distinguish the LS and other familial colorectal cancer (CRC). We recruited 132 patients clinically suspected LS. All patients fulfilled at least one criterion of the revised Bethesda guidelines (RBG), while only 45% met the more stringent Amsterdam Criteria II. (ACII). Previous studies have emphasized that the ACII, with its higher specificity, is more effective for the detection of LS, whereas the RBG offers high sensitivity and is therefore more suitable as a screening tool for selection of individuals for further molecular testing [41,42]. Although all probands fulfilled at least one criterion of the RBG, we could detect germline PVs/LPVs and promising VUSs in *MLH1*, *MSH2*, and *PMS2* in only 20% of the probands. This detection rate appears lower than some prior enriched cohorts but higher than population-based estimates [43–45]. Because our cohort was limited and clinically enriched, the results cannot be interpreted as reflecting the prevalence of LS in Southern Thai population. Larger population-based study in Thailand will be necessary to clarify the true frequency of LS in Thailand and to determine whether the RBG or alternative strategies provide the best balance of sensitivity and specificity in this context.

In this study, MSI testing was performed in parallel with the high-throughput sequencing for research purposes. None of the 43 patients with MSS carried a germline MMR PV, whereas 13 of the 18 MSI-H patients (72%) did. These findings demonstrate that MSI testing and/or MMR IHC substantially improve diagnostic yield and help avoid unnecessary genetic testing. In particular, omitting MMR gene sequencing in clearly MSS cases may reduce overall diagnostic costs. As a tumor-based screening strategy, MSI testing and MMR IHC can be applied either to patients meeting the RBG, or, in some institutions, to all CRC or EC cases. Beyond identifying LS-related cancers, these tests also provide prognostic and therapeutic information, as MSI-H/dMMR CRCs show better survival and may benefit from immune checkpoint inhibitors [46,47]. Despite well-established LS diagnostic guidelines in high-income countries, implementation in Thailand remains limited, primarily due to financial constraints and insufficient hospital availability. [3,48,49]. The shortage of surgeons and genetic counsellors, together with the practice of initiating CRC screening at age 50 years [3,23,50,51], further impedes early detection. Given the high cost and limited availability of diagnostic and preventive services, careful evaluation using the RBG and/or the ACII remains the most efficient strategy for identifying individual at risk of LS in resource-limited settings [52,53].

Although an *MLH1* germline variant is the most common finding in LS, followed by *MSH2*, our study differs in that the number of *MLH1* carriers was equal to that of *MSH2* carriers (46% each), while the remaining 8% carried a *PMS2* PV. We did not observe any germline PV/LPV in *MSH6* and *EPCAM* which is unsurprising because its prevalence is extremely low among these genes [54,55]. None of SV PV/LPV in the MMR genes was detected.

According to previous reports, the average age of onset of cancers is typically ≤ 45 years [42,45,56]. The average age of onset of cancer in our study was 52 years, which is slightly higher than earlier reports but aligns with the report of De la Chapelle and colleagues, that LS can be detected in individuals older than 50 years [57]. Our data indicate that EC tends to occur at a younger age (mean 48 years) than CRC (mean 52 years). Age-stratified analysis showed that the majority of patients (70%) were diagnosed before age 60, whereas only 8% developed cancer after age 70. We also observed notable gene-specific patterns. *MLH1* PV carriers developed cancer at a mean age of 50 years, whereas *MSH2* carriers were diagnosed earlier, at an average of 44 years. Moreover, *MSH2* carriers exhibited a higher proportion of extracolonic malignancies (42%) and a lower proportion of CRC (67%) compared with *MLH1* carriers (33% and 92%, respectively) [58,59]. These findings are consistent with previous reports describing broader tumor spectra and higher extracolonic cancer burdens associated spectra and higher extracolonic cancer burdens associated with *MSH2* variants.

No difference in frequency was observed in right- versus left-sided CRC among LS patients carrying a PV in DNA MMR genes. Baran and colleagues previously reported that proximal (right-sided) colon tumor is more related to the DNA MMR pathway, whereas distal (left-sided) colon tumor is more often related to the chromosomal instability pathway [60]. However, among the 122 CRC patients suspected of LS in our study, more than 70% presented with left-sided tumors. The average age of onset did not differ between tumor locations: 49.7 ± 11.6 years for left-sided and 53.5 ± 14.2 years for right-sided tumors. These findings suggest that, in our population, tumor laterality may not be a distinguishing feature of LS-associated CRC.

Regarding EC, 13 of the 132 suspected LS patients developed EC. Two of them developed EC and OC at the same age of onset, three had both EC and CRC, and eight had EC alone. Our study found PVs/LPVs in MMR genes in six EC patients (46%). This number is higher than previous studies in Thai cohorts which indicated that the prevalence of LS among EC patients has been estimated at approximately 35% [21,61]. This higher detection rate may reflect differences in referral criteria, population characteristics, or the inclusion of multi-cancer presentations in our study.

For the *MSH2* c.1237T > C, identified in one family (Fig 1), we estimated a penetrance rate of 56% based on the proportion of carriers who developed cancer. Previous studies have reported wide variability in the penetrance of CRC and EC among LS patients, ranging from 30–80% [62]. Because cancer risk tends to increase with age, the actual penetrance could be underestimated in younger individuals who may develop the disease later in life. Moreover, polyps were found in two individuals during surveillance. Without timely intervention, these lesions may have progressed to CRC, further suggesting that the true penetrance may be higher. This observation aligns with prior literature indicating that LS exhibits high but incomplete penetrance, reaching up to 85% in some reports [54,59,63].

In our study, the cumulative risk of CRC or EC increased with age and was higher among female than male carriers of *MSH2* variant. This pattern differs from previous studies which report similar of higher risks in males [64,65]. The earlier onset in females may reflect the EC’s contribution, which typically presents at a younger age than CRC. These observations emphasize the importance of early screening, particularly for females before age 50, while prolonged surveillance may be warranted in male carriers.

Due to the absence of routine hereditary cancer screening in Thailand, many cases likely remain undiagnosed until later stages, leading to an underestimation of disease prevalence. Establishing comprehensive genetic testing and surveillance guidelines would enable earlier detection and improve clinical outcomes in high-risk individuals. Long-term follow-up remains essential to accurately assess lifetime cancer risk.

The tightly knit communities of Southern Thai populations facilitate reconstruction of extended familial pedigrees, allowing genealogical tracing up to six generations. This demographic structure enhances the ability to track hereditary patterns and recruit at-risk relatives who may benefit from genetic counseling and targeted screening. Consistent with our earlier study, we traced back to four generations and confirmed the pathogenicity of the *APC* germline variant in a southern Thai family with familial adenomatous polyposis (FAP) [66], the present findings reinforce the value of co-segregation analysis for validating variants and guiding clinical decision-making. Integrating genetic counseling into public health initiatives is crucial for raising awareness of hereditary cancers, promoting early testing, and encouraging preventive measures. This is particularly important for hereditary CRC, where penetrance is high and early intervention can substantially reduce cancer incidence [50,66]. Ultimately, early detection and prevention improve survival outcomes, quality of life, and healthcare cost-effectiveness.

Our study demonstrates the economic value of integrating genetic testing into Lynch syndrome management. By linking the identification of probands and cascade testing of relatives with cost data from Thailand, we showed that genetic testing achieves a benefit–cost ratio (BCR = 3.39). Considering that the implementation of panel testing is expected to reduce the per-case cost of genetic testing by approximately 60% (estimated 20,000 THB per proband). This strategy could yield a substantial direct cost advantage, resulting in an approximately 72% reduction compared with endoscopy-based surveillance. The resulting BCR of 9.41 indicates that each 1 THB invested generate 9.41 THB in returns through avoided surveillance and treatment expenditures. Our findings reported evidence supporting the cost-effectiveness of investment in the testing, which is justified when compared with the expenses associated with unnecessary endoscopic procedures in unaffected individuals. In brief, the test becomes more cost-effective as the number of at-risk members in the family increases. In addition, it also yields the same direction as the previous study in breast cancer patients and family members in Thailand which led to the national health benefits package in 2022 [49,67]. For this reason, we would reinforce how genetic counseling is useful for the prevention of hereditary cancer and that the authorities would benefit from implementing it in the national benefit package policy.

## Conclusions

Hereditary colorectal cancer exhibits a clear pattern of inheritance and high penetrance. Through the Genomics Thailand Project, germline pathogenic variants were identified in a large local cohort. Genetic counseling and segregation analysis confirmed a shared ancestry consistent with an extended kindred. Predictive testing enabled the identification of at-risk family members, facilitating targeted cancer prevention. This study, one of the first in Thai patients with suspected Lynch syndrome, demonstrates the effectiveness of family-based genetic counseling in cancer prevention and informs risk-adapted surveillance strategies. The findings emphasize the need to integrate genetic counseling and surveillance into routine care and support the consideration of nationwide genetic testing policies in Thailand.

## Supporting information

S1 Table Quality data of 132 WGSs specified in *EPCAM, MSH2, MSH6, MLH1,* and *PMS2.* (XLSX)

S2 Table Structural Variants of 132 patients found in *MSH2* and *MLH1.* (XLSX)

S3 Table Identity-by-descent results show relatedness among 132 patients. (XLSX)

## References

[1] Bray F, Laversanne M, Sung H, Ferlay J, Siegel RL, Soerjomataram I. Global cancer statistics 2022: GLOBOCAN estimates of incidence and mortality worldwide for 36 cancers in 185 countries. CA Cancer J Clin. 2024;74(3):229–63. 38572751 10.3322/caac.21834

[2] Phimha S, Promthet S, Suwanrungruang K, Chindaprasirt J, Bouphan P, Santong C, et al. Health insurance and colorectal cancer survival in Khon Kaen, Thailand. Asian Pac J Cancer Prev. 2019;20(6):1797–802. doi: 10.31557/APJCP.2019.20.6.1797 31244302 PMC7021590

[3] Lohsiriwat V, Chaisomboon N, Pattana-Arun J. Current colorectal cancer in Thailand. Ann Coloproctol. 2020;36(2):78–82. 32054248 10.3393/ac.2020.01.07 PMC7299565

[4] Fearon ER. Molecular genetics of colorectal cancer. Annu Rev Pathol. 2011;6:479–507. doi: 10.1146/annurev-pathol-011110-130235 21090969

[5] Hegde M, Ferber M, Mao R, Samowitz W, Ganguly A, Working Group of the American College of Medical Genetics and Genomics (ACMG) Laboratory Quality Assurance Committee. ACMG technical standards and guidelines for genetic testing for inherited colorectal cancer (Lynch syndrome, familial adenomatous polyposis, and MYH-associated polyposis). Genet Med. 2014;16(1):101–16. doi: 10.1038/gim.2013.166 24310308

[6] Cerretelli G, Ager A, Arends MJ, Frayling IM. Molecular pathology of Lynch syndrome. J Pathol. 2020;250(5):518–31. doi: 10.1002/path.5422 32141610

[7] Dominguez-Valentin M, Sampson JR, Seppälä TT, Ten Broeke SW, Plazzer J-P, Nakken S, et al. Correction: Cancer risks by gene, age, and gender in 6350 carriers of pathogenic mismatch repair variants: findings from the Prospective Lynch Syndrome Database. Genet Med. 2020;22(9):1569. doi: 10.1038/s41436-020-0892-4 32690931 PMC7462742

[8] Møller P, Seppälä TT, Ahadova A, Crosbie EJ, Holinski-Feder E, Scott R, et al. Dominantly inherited micro-satellite instable cancer - the four Lynch syndromes - an EHTG, PLSD position statement. Hered Cancer Clin Pract. 2023;21(1):19. doi: 10.1186/s13053-023-00263-3 37821984 PMC10568908

[9] Silinskaite U, Gavelienė E, Stulpinas R, Janavicius R, Poskus T. A novel mutation of MSH2 gene in a patient with Lynch syndrome presenting with thirteen metachronous malignancies. J Clin Med. 2023;12(17):5502. doi: 10.3390/jcm12175502 37685569 PMC10488139

[10] Ahadova A, Gallon R, Gebert J, Ballhausen A, Endris V, Kirchner M, et al. Three molecular pathways model colorectal carcinogenesis in Lynch syndrome. Int J Cancer. 2018;143(1):139–50. doi: 10.1002/ijc.31300 29424427

[11] Suerink M, Rodríguez-Girondo M, van der Klift HM, Colas C, Brugieres L, Lavoine N, et al. An alternative approach to establishing unbiased colorectal cancer risk estimation in Lynch syndrome. Genet Med. 2019;21(12):2706–12. doi: 10.1038/s41436-019-0577-z 31204389

[12] Umar A, Boland CR, Terdiman JP, Syngal S, de la Chapelle A, Rüschoff J, et al. Revised Bethesda Guidelines for hereditary nonpolyposis colorectal cancer (Lynch syndrome) and microsatellite instability. J Natl Cancer Inst. 2004;96(4):261–8. doi: 10.1093/jnci/djh034 14970275 PMC2933058

[13] Vasen HF, Watson P, Mecklin JP, Lynch HT. New clinical criteria for hereditary nonpolyposis colorectal cancer (HNPCC, Lynch syndrome) proposed by the International Collaborative group on HNPCC. Gastroenterology. 1999;116(6):1453–6. doi: 10.1016/s0016-5085(99)70510-x 10348829

[14] Edwards P, Monahan KJ. Diagnosis and management of Lynch syndrome. Frontline Gastroenterol. 2022;13(e1):e80–7. doi: 10.1136/flgastro-2022-102123 35812033 PMC9234730

[15] Hampel H, Frankel WL, Martin E, Arnold M, Khanduja K, Kuebler P, et al. Feasibility of screening for Lynch syndrome among patients with colorectal cancer. J Clin Oncol. 2008;26(35):5783–8. doi: 10.1200/JCO.2008.17.5950 18809606 PMC2645108

[16] Yurgelun MB, Kulke MH, Fuchs CS, Allen BA, Uno H, Hornick JL, et al. Cancer susceptibility gene mutations in individuals with colorectal cancer. J Clin Oncol. 2017;35(10):1086–95. doi: 10.1200/JCO.2016.71.0012 28135145 PMC5455355

[17] Ryan NAJ, Glaire MA, Blake D, Cabrera-Dandy M, Evans DG, Crosbie EJ. The proportion of endometrial cancers associated with Lynch syndrome: a systematic review of the literature and meta-analysis. Genet Med. 2019;21(10):2167–80. doi: 10.1038/s41436-019-0536-8 31086306 PMC8076013

[18] Hampel H, Frankel WL, Martin E, Arnold M, Khanduja K, Kuebler P, et al. Screening for the Lynch syndrome (hereditary nonpolyposis colorectal cancer). N Engl J Med. 2005;352(18):1851–60. doi: 10.1056/NEJMoa043146 15872200

[19] Lamberti C, Mangold E, Pagenstecher C, Jungck M, Schwering D, Bollmann M, et al. Frequency of hereditary non-polyposis colorectal cancer among unselected patients with colorectal cancer in Germany. Digestion. 2006;74(1):58–67. doi: 10.1159/000096868 17095871

[20] Lynch HT, Lynch PM, Lanspa SJ, Snyder CL, Lynch JF, Boland CR. Review of the Lynch syndrome: history, molecular genetics, screening, differential diagnosis, and medicolegal ramifications. Clin Genet. 2009;76(1):1–18. doi: 10.1111/j.1399-0004.2009.01230.x 19659756 PMC2846640

[21] Atjimakul T, Wattanapaisal P, Suwiwat S, Wanichsuwan W, Hanprasertpong J. Microsatellite instability and oncological outcomes in Thai patients with endometrial cancer. J Obstet Gynaecol. 2022;42(7):3117–23. doi: 10.1080/01443615.2022.2106557 35930016

[22] Shotelersuk V, Tongsima S, Pithukpakorn M, Eu-Ahsunthornwattana J, Mahasirimongkol S. Precision medicine in Thailand. Am J Med Genet C Semin Med Genet. 2019;181(2):245–53. 30888117 10.1002/ajmg.c.31694

[23] Kittikoon S, Pithukpakorn M, Pramyothin P. Physician awareness, preparedness, and opinions toward consumer-initiated genetic testing in Thailand: views from a changing landscape. J Genet Couns. 2021;30(6):1535–43. doi: 10.1002/jgc4.1420 33931918

[24] Samaison L, Grall M, Staroz F, Uguen A. Microsatellite instability diagnosis using the fully automated Idylla platform: feasibility study of an in-house rapid molecular testing ancillary to immunohistochemistry in pathology laboratories. J Clin Pathol. 2019;72(12):830–5. doi: 10.1136/jclinpath-2019-205935 31235541

[25] Li H, Durbin R. Fast and accurate short read alignment with Burrows-Wheeler transform. Bioinformatics. 2009;25(14):1754–60. doi: 10.1093/bioinformatics/btp324 19451168 PMC2705234

[26] van der Auwera G, O’Connor BD. Genomics in the cloud: using Docker, GATK, and WDL in Terra. O’Reilly Media, Incorporated; 2020.

[27] McLaren W, Gil L, Hunt SE, Riat HS, Ritchie GR, Thormann A. The ensembl variant effect predictor. Genome Biol. 2016;17(1):122. 27268795 10.1186/s13059-016-0974-4 PMC4893825

[28] Liu X, Li C, Mou C, Dong Y, Tu Y. dbNSFP v4: a comprehensive database of transcript-specific functional predictions and annotations for human nonsynonymous and splice-site SNVs. Genome Med. 2020;12(1):103. doi: 10.1186/s13073-020-00803-9 33261662 PMC7709417

[29] Chen S, Francioli LC, Goodrich JK, Collins RL, Kanai M, Wang Q, et al. A genomic mutational constraint map using variation in 76,156 human genomes. Nature. 2024;625(7993):92–100. doi: 10.1038/s41586-023-06045-0 38057664 PMC11629659

[30] Xavier A, Scott RJ, Talseth-Palmer BA. TAPES: a tool for assessment and prioritisation in exome studies. PLoS Comput Biol. 2019;15(10):e1007453. doi: 10.1371/journal.pcbi.1007453 31613886 PMC6814239

[31] Richards S, Aziz N, Bale S, Bick D, Das S, Gastier-Foster J, et al. Standards and guidelines for the interpretation of sequence variants: a joint consensus recommendation of the American College of Medical Genetics and Genomics and the Association for Molecular Pathology. Genet Med. 2015;17(5):405–24. doi: 10.1038/gim.2015.30 25741868 PMC4544753

[32] Chen X, Schulz-Trieglaff O, Shaw R, Barnes B, Schlesinger F, Källberg M, et al. Manta: rapid detection of structural variants and indels for germline and cancer sequencing applications. Bioinformatics. 2016;32(8):1220–2. doi: 10.1093/bioinformatics/btv710 26647377

[33] Geoffroy V, Herenger Y, Kress A, Stoetzel C, Piton A, Dollfus H, et al. AnnotSV: an integrated tool for structural variations annotation. Bioinformatics. 2018;34(20):3572–4. doi: 10.1093/bioinformatics/bty304 29669011

[34] Manichaikul A, Mychaleckyj JC, Rich SS, Daly K, Sale M, Chen W-M. Robust relationship inference in genome-wide association studies. Bioinformatics. 2010;26(22):2867–73. doi: 10.1093/bioinformatics/btq559 20926424 PMC3025716

[35] Claus EB, Risch N, Thompson WD. Genetic analysis of breast cancer in the cancer and steroid hormone study. Am J Hum Genet. 1991;48(2):232–42. 1990835 PMC1683001

[36] de Jong AE, Vasen HFA. The frequency of a positive family history for colorectal cancer: a population-based study in the Netherlands. Neth J Med. 2006;64(10):367–70. 17122453

[37] Quehenberger F, Vasen HFA, van Houwelingen HC. Risk of colorectal and endometrial cancer for carriers of mutations of the hMLH1 and hMSH2 gene: correction for ascertainment. J Med Genet. 2005;42(6):491–6. doi: 10.1136/jmg.2004.024299 15937084 PMC1736072

[38] Breast Cancer Association C, Dorling L, Carvalho S, Allen J, Gonzalez-Neira A, Luccarini C, et al. Breast cancer risk genes - association analysis in more than 113,000 women. N Engl J Med. 2021;384(5):428–39. 33471991 10.1056/NEJMoa1913948 PMC7611105

[39] Wanitsuwan W, Boonpipatanapong T, Kanngern S, Chaiyapan W, Graidist P, Sangkhathat S. Clinical report. Benefits from genetic test in descendants of familial adenomatous polyposis syndrome: report of a family in Southern Thailand. Asian Biomed. 2011;5(4):553–7. doi: 10.5372/1905-7415.0504.074

[40] Bhimani N, Wong GYM, Molloy C, Pavlakis N, Diakos CI, Clarke SJ, et al. Cost of treating metastatic colorectal cancer: a systematic review. Public Health. 2022;211:97–104. doi: 10.1016/j.puhe.2022.06.022 36063775

[41] Thavornpattanapong T, Kanjanapradit K, Sangkhathat S, Wanitsuwan W. The effectiveness of clinical guidelines in the diagnosis of Lynch syndrome compared to microsatellite instability and immunohistochemistry analyses in southern Thailand. J Health Sci Med Res. 2019. doi: 10.31584/jhsmr.201938

[42] Bellizzi AM, Frankel WL. Colorectal cancer due to deficiency in DNA mismatch repair function: a review. Adv Anat Pathol. 2009;16(6):405–17. doi: 10.1097/PAP.0b013e3181bb6bdc 19851131

[43] Fanale D, Corsini LR, Brando C, Dimino A, Filorizzo C, Magrin L, et al. Impact of different selection approaches for identifying Lynch syndrome-related colorectal cancer patients: unity is strength. Front Oncol. 2022;12:827822. doi: 10.3389/fonc.2022.827822 35223509 PMC8864140

[44] Moreira L, Balaguer F, Lindor N, de la Chapelle A, Hampel H, Aaltonen LA, et al. Identification of Lynch syndrome among patients with colorectal cancer. JAMA. 2012;308(15):1555–65. doi: 10.1001/jama.2012.13088 23073952 PMC3873721

[45] Peltomaki P, Nystrom M, Mecklin JP, Seppala TT. Lynch syndrome genetics and clinical implications. Gastroenterology. 2023;164(5):783–99. 36706841 10.1053/j.gastro.2022.08.058

[46] Popat S, Hubner R, Houlston RS. Systematic review of microsatellite instability and colorectal cancer prognosis. J Clin Oncol. 2005;23(3):609–18. doi: 10.1200/JCO.2005.01.086 15659508

[47] Sahin IH, Akce M, Alese O, Shaib W, Lesinski GB, El-Rayes B, et al. Immune checkpoint inhibitors for the treatment of MSI-H/MMR-D colorectal cancer and a perspective on resistance mechanisms. Br J Cancer. 2019;121(10):809–18. doi: 10.1038/s41416-019-0599-y 31607751 PMC6889302

[48] Horpaopan SSJ. The utility of histopathological features predicting microsatellite high (MSI-H) colorectal cancer in a limited setting. Int J Clin Exp Med. 2020;13(3):1878–87.

[49] Lertwilaiwittaya P, Tantai N, Maneeon S, Kongbunrak S, Nonpanya N, Hurst ACE, et al. A cost-utility analysis of BRCA1 and BRCA2 testing in high-risk breast cancer patients and family members in Thailand: a cost-effective policy in resource-limited settings. Front Public Health. 2023;11:1257668. doi: 10.3389/fpubh.2023.1257668 38162618 PMC10757601

[50] Laurino MY, Leppig KA, Abad PJ, Cham B, Chu YWY, Kejriwal S. A report on ten Asia Pacific countries on current status and future directions of the genetic counseling profession: the establishment of the professional society of genetic counselors in Asia. J Genet Couns. 2018;27(1):21–32. 28699126 10.1007/s10897-017-0115-6

[51] Cutiongco-de la Paz EM, Chung BH-Y, Faradz SMH, Thong M-K, David-Padilla C, Lai PS, et al. Training in clinical genetics and genetic counseling in Asia. Am J Med Genet C Semin Med Genet. 2019;181(2):177–86. doi: 10.1002/ajmg.c.31703 31037827

[52] Quezada-Diaz F, Gomez J, Fulle A, Irarrazaval MJ, Torres J, Bellolio F. Results of an immunohistochemistry-based universal screening strategy for Lynch syndrome in patients with colorectal cancer treated in a public hospital from Latin-America. Surg Open Dig Adv. 2022;5:100041. doi: 10.1016/j.soda.2022.100041

[53] Tognetto A, Michelazzo MB, Calabró GE, Unim B, Di Marco M, Ricciardi W, et al. A systematic review on the existing screening pathways for Lynch syndrome identification. Front Public Health. 2017;5:243. doi: 10.3389/fpubh.2017.00243 28955708 PMC5600943

[54] Silva FCC da, Valentin MD, Ferreira F de O, Carraro DM, Rossi BM. Mismatch repair genes in Lynch syndrome: a review. Sao Paulo Med J. 2009;127(1):46–51. doi: 10.1590/s1516-31802009000100010 19466295 PMC10969316

[55] Chen E, Xu X, Liu T. Hereditary nonpolyposis colorectal cancer and cancer syndromes: recent basic and clinical discoveries. J Oncol. 2018;2018:3979135. 29849630 10.1155/2018/3979135 PMC5937448

[56] Lynch HT, Boland CR, Gong G, Shaw TG, Lynch PM, Fodde R, et al. Phenotypic and genotypic heterogeneity in the Lynch syndrome: diagnostic, surveillance and management implications. Eur J Hum Genet. 2006;14(4):390–402. doi: 10.1038/sj.ejhg.5201584 16479259

[57] de la Chapelle A, Palomaki G, Hampel H. Identifying Lynch syndrome. Int J Cancer. 2009;125(6):1492–3. 19536819 10.1002/ijc.24491 PMC2771416

[58] Vasen HF, Stormorken A, Menko FH, Nagengast FM, Kleibeuker JH, Griffioen G, et al. MSH2 mutation carriers are at higher risk of cancer than MLH1 mutation carriers: a study of hereditary nonpolyposis colorectal cancer families. J Clin Oncol. 2001;19(20):4074–80. doi: 10.1200/JCO.2001.19.20.4074 11600610

[59] Sehgal R, Sheahan K, O’Connell PR, Hanly AM, Martin ST, Winter DC. Lynch syndrome: an updated review. Genes (Basel). 2014;5(3):497–507. doi: 10.3390/genes5030497 24978665 PMC4198913

[60] Baran B, Mert Ozupek N, Yerli Tetik N, Acar E, Bekcioglu O, Baskin Y. Difference between left-sided and right-sided colorectal cancer: a focused review of literature. Gastroenterology Res. 2018;11(4):264–73. 30116425 10.14740/gr1062w PMC6089587

[61] Manchana T, Ariyasriwatana C, Triratanachat S, Phowthongkum P. Lynch syndrome in Thai endometrial cancer patients. Asian Pac J Cancer Prev. 2021;22(5):1477–83. doi: 10.31557/APJCP.2021.22.5.1477 34048176 PMC8408393

[62] Antelo M, Golubicki M, Roca E, Mendez G, Carballido M, Iseas S, et al. Lynch-like syndrome is as frequent as Lynch syndrome in early-onset nonfamilial nonpolyposis colorectal cancer. Int J Cancer. 2019;145(3):705–13. doi: 10.1002/ijc.32160 30693488 PMC10423080

[63] Win AK, Jenkins MA, Dowty JG, Antoniou AC, Lee A, Giles GG, et al. Prevalence and penetrance of major genes and polygenes for colorectal cancer. Cancer Epidemiol Biomarkers Prev. 2017;26(3):404–12. doi: 10.1158/1055-9965.EPI-16-0693 27799157 PMC5336409

[64] Hodan R, Gupta S, Weiss JM, Axell L, Burke CA, Chen LM. Genetic/familial high-risk assessment: colorectal, endometrial, and gastric, version 3.2024, NCCN clinical practice guidelines in oncology. J Natl Compr Canc Netw. 2024;22(10):695–711. 39689429 10.6004/jnccn.2024.0061

[65] Gonzalez-Flores E, Garcia-Carbonero R, Elez E, Redondo-Cerezo E, Safont MJ, Vera Garcia R. Gender and sex differences in colorectal cancer screening, diagnosis and treatment. Clin Transl Oncol. 2025. 10.1007/s12094-024-03801-0 PMC12178976 39821481

[66] Wanitsuwan W, Vijasika S, Jirarattanasopa P, Horpaopan S. A distinct APC pathogenic germline variant identified in a southern Thai family with familial adenomatous polyposis. BMC Med Genomics. 2021;14(1):87. doi: 10.1186/s12920-021-00933-y 33740971 PMC7980625

[67] Kwong A, Tan DS-P, Ryu JM, ACROSS Consortium. Current practices and challenges in genetic testing and counseling for women with breast and ovarian cancer in Asia. Asia Pac J Clin Oncol. 2025;21(2):211–20. doi: 10.1111/ajco.14074 38776249 PMC11880987

